# Interventions for improving mobility after hip fracture surgery in adults

**DOI:** 10.1590/S1516-31802011000600012

**Published:** 2011-12-01

## Abstract

**BACKGROUND:**

Hip fracture mainly occurs in older people. Strategies to improve mobility include gait retraining, various forms of exercise and muscle stimulation.

**OBJECTIVES:**

To evaluate the effects of different interventions for improving mobility after hip fracture surgery in adults.

**SEARCH STRATEGY:**

We searched the Cochrane Bone, Joint and Muscle Trauma Group Specialised Register, the Cochrane Central Register of Controlled Trials, MEDLINE and other databases, and reference lists of articles, up to April 2010.

**SELECTION CRITERIA:**

All randomised or quasi-randomised trials comparing different mobilisation strategies after hip fracture surgery.

**DATA COLLECTION AND ANALYSIS:**

The authors independently selected trials, assessed risk of bias and extracted data. There was no data pooling.

**MAIN RESULTS:**

The 19 included trials (involving 1589 older adults) were small, often with methodological flaws. Just two pairs of trials tested similar interventions. Twelve trials evaluated mobilisation strategies started soon after hip fracture surgery. Single trials found improved mobility from, respectively, a two-week weight-bearing programme, a quadriceps muscle strengthening exercise programme and electrical stimulation aimed at alleviating pain. Single trials found no significant improvement in mobility from, respectively, a treadmill gait retraining programme, 12 weeks of resistance training, and 16 weeks of weight-bearing exercise. One trial testing ambulation started within 48 hours of surgery found contradictory results. One historic trial found no significant difference in unfavourable outcomes for weight bearing started at two versus 12 weeks. Of two trials evaluating more intensive physiotherapy regimens, one found no difference in recovery, the other reported a higher level of drop-out in the more intensive group. Two trials tested electrical stimulation of the quadriceps: one found no benefit and poor tolerance of the intervention; the other found improved mobility and good tolerance. Seven trials evaluated strategies started after hospital discharge. Started soon after discharge, two trials found improved outcome after 12 weeks of intensive physical training and a home-based physical therapy programme respectively. Begun after completion of standard physical therapy, one trial found improved outcome after six months of intensive physical training, one trial found increased activity levels from a one year exercise programme, and one trial found no significant effects of home-based resistance or aerobic training. One trial found improved outcome after home-based exercises started around 22 weeks from injury. One trial found home-based weight-bearing exercises starting at seven months produced no significant improvement in mobility.

**AUTHORS’ CONCLUSIONS:**

There is insufficient evidence from randomised trials to establish the best strategies for enhancing mobility after hip fracture surgery.

Titular Professor and Head of the Department of Orthopedics and Traumatology, Faculdade de Medicina da Universidade de São Paulo (FMUSP), São Paulo, Brazil.

**This review was published in Cochrane Database of Systematic Reviews 2011, Issue 3. Art. No.:** CD001704. DOI: 10.1002/14651858.CD001704.pub4. For full details, see reference 1.

**The full text of this review is available (open access) from:** http://www.cochranejournalclub.com/interventions-improving-mobility-hip-fracture-clinical/pdf/CD001704_abstract.pdf

**This section was edited under the responsibility of the Brazilian Cochrane Center – www.centrocochranedobrasil.org**

## COMMENTS

A meta-analysis is always an important paper, but the main purpose of this paper should be the early and late complications concerning this procedure and not different methods of postoperative rehabilitation.
